# Survival Analysis of Long-Term Exposure to Different Sizes of Airborne Particulate Matter and Risk of Infant Mortality Using a Birth Cohort in Seoul, Korea

**DOI:** 10.1289/ehp.1002364

**Published:** 2010-12-17

**Authors:** Ji-Young Son, Michelle L. Bell, Jong-Tae Lee

**Affiliations:** 1 School of Forestry and Environmental Studies, Yale University, New Haven, Connecticut, USA; 2 Department of Environmental Health, College of Health Science, Korea University, Seoul, Korea

**Keywords:** air pollution, Cox proportional hazards model, infant mortality, long-term effect, particulate matter, PM_2.5_, PM_10_, PM_10–2.5_, survival analysis, time dependent, TSP

## Abstract

**Background:**

Several studies suggest that airborne particulate matter (PM) is associated with infant mortality; however, most focused on short-term exposure to larger particles.

**Objectives:**

We evaluated associations between long-term exposure to different sizes of particles [total suspended particles (TSP), PM ≤ 10 μm in aerodynamic diameter (PM_10_), ≤ 10–2.5 μm (PM_10–2.5_), and ≤ 2.5 μm (PM_2.5_)] and infant mortality in a cohort in Seoul, Korea, 2004–2007.

**Methods:**

The study includes 359,459 births with 225 deaths. We applied extended Cox proportional hazards modeling with time-dependent covariates to three mortality categories: all causes, respiratory, and sudden infant death syndrome (SIDS). We calculated exposures from birth to death (or end of eligibility for outcome at 1 year of age) and pregnancy (gestation and each trimester) and treated exposures as time-dependent variables for subjects’ exposure for each pollutant. We adjusted by sex, gestational length, season of birth, maternal age and educational level, and heat index. Each cause of death and exposure time frame was analyzed separately.

**Results:**

We found a relationship between gestational exposures to PM and infant mortality from all causes or respiratory causes for normal-birth-weight infants. For total mortality (all causes), risks were 1.44 (95% confidence interval, 1.06–1.97), 1.65 (1.18–2.31), 1.53 (1.22–1.90), and 1.19 (0.83–1.70) per interquartile range increase in TSP, PM_10_, PM_2.5_, and PM_10–2.5_, respectively; for respiratory mortality, risks were 3.78 (1.18–12.13), 6.20 (1.50–25.66), 3.15 (1.26–7.85), and 2.86 (0.76–10.85). For SIDS, risks were 0.92 (0.33–2.58), 1.15 (0.38–3.48), 1.42 (0.71–2.87), and 0.57 (0.16–1.96), respectively.

**Conclusions:**

Our findings provide supportive evidence of an association of long-term exposure to PM air pollution with infant mortality.

Numerous epidemiologic studies have demonstrated associations between ambient air pollution and health outcomes, including mortality (Chen et al. 2008; Dales et al. 2004; Qian et al. 2007), hospitalizations (Dominici et al. 2006; Lin et al. 2004; Villeneuve et al. 2007; Wellenius et al. 2005), and lung function (Delfino et al. 2008; Jalaludin et al. 2000; Tang et al. 2007). Infant mortality is still a major contributor to childhood mortality (Glinianaia et al. 2004a). Infants and children are potentially susceptible because of their young immune systems, developing respiratory and other systems, and common viral infections (Bateson and Schwartz 2008; Glinianaia et al. 2004b; Koranteng et al. 2007).

Findings for air pollution and infant health are relatively consistent for particulate matter (PM) compared with other pollutants (Šrám et al. 2005). Several studies suggest that PM exposure is associated with infant mortality (Bobak and Leon 1999; Ha et al. 2003; Hajat et al. 2007; Lipfert et al. 2000; Romieu et al. 2004; Tsai et al. 2006; Woodruff et al. 1997). However, most studies focused on short-term exposure to larger particles such as total suspended particulate (TSP) or PM_10_ (PM ≤ 10 μm in aerodynamic diameter) and to exposures that occurred postneonatally. Research on effects of PM_2.5_ (≤ 2.5 μm) or coarse particles PM_10–2.5_ (2.5–10 μm) in infants is limited.

Only two studies evaluated associations between long-term PM_2.5_ exposure and infant mortality. Woodruff et al. (2006) observed an association between postneonatal respiratory mortality and PM_2.5_ exposure from birth to death in California. Another U.S. study did not find a relationship between PM_2.5_ during the first 2 months of life and infant mortality (Woodruff et al. 2008).

Smaller particles (PM_2.5_) may be more harmful than larger particles because they consist of different chemical components, with more combustion-related sources. Smaller particles penetrate more deeply into lung airways and are deposited in the alveolar region more often. Few studies have evaluated effects of particle size on infant mortality, although many studies in adults suggest that PM_2.5_ is more strongly associated with health than is PM_10_ (Franklin et al. 2007).

Relatively few studies have evaluated air pollution and infant mortality in Korea (Ha et al. 2003; Son et al. 2008). Moreover, these studies focused on short-term PM_10_ exposure postneonatally. Also, no study investigated the relationship between exposure to air pollution during pregnancy and infant mortality. We evaluated associations between long-term exposure (during pregnancy, and from birth to death or end of eligibility for outcome at 1 year of age) to different particle sizes (TSP, PM_10_, PM_10–2.5_, PM_2.5_) and infant mortality in a birth cohort in Seoul, Korea, for 2004–2007. We used an extended Cox proportional hazard model designed to assess long-term effects of PM while estimating cumulative lifetime exposure and average exposure during pregnancy as time-dependent variables.

## Materials and Methods

### Health data

We obtained linked birth and mortality records for 2004–2007 from the Korean National Statistical Office for 381,271 subjects. Birth data included reported residential address at birth, mother’s permanent residential address, sex, birth weight (grams), parents’ age (years), parents’ education (none; elementary, middle, high school; more than university; unknown), parents’ occupation (manager, expert, engineer, office worker, service job, salesperson, agriculture/fishery/forestry, technical service, mechanic, physical labor, student/unemployed/household, unknown/military), parity, birth order, gestational age (weeks), parents’ marital status (yes, no, unknown), birth month, and place of birth (home, hospital, other, unknown). Mortality data included infant’s residential address, date of death, place of death (e.g., home, hospital), sex, age, and primary and secondary causes of death.

We excluded subjects whose reported residential addresses at birth differed from the mother’s permanent residential addresses on birth certificates and subjects with different residential addresses for birth and death. For study subjects who survived, residential addresses are available only at birth. We excluded observations with incomplete birth certificate data for infant’s sex, gestational length, birth weight, and mother’s education or age. Study subjects were restricted to infants with 37–44 weeks of gestation. These criteria resulted in a loss of 5.7% of observations. To consider only deaths potentially associated with air pollution, we omitted infants who died in the neonatal period (< 28 days) because these deaths are more likely to occur in infants who had not left the hospital after birth or experienced pregnancy-related complications (Woodruff et al. 2006). After exclusions, 359,459 subjects were included. The distribution of values for variables used for analysis was similar between study subjects included in analysis and all subjects.

We classified mortality data by cause of death according to the *International Classification of Diseases, 10th Revision* (ICD-10) (World Health Organization 1993), from death certificate information in linked birth and mortality records. We considered total mortality as all causes of death except external causes (ICD-10 codes A00–R99), sudden infant death syndrome (SIDS; ICD-10 R95), and respiratory causes (ICD-10 J00–J99). The primary cause of death on death certificates was used.

### Air pollution and meteorologic data

TSP, PM_10_, and PM_2.5_ monitoring data were obtained from 27 monitoring stations distributed evenly throughout Seoul and operated by the Department of Environment, Republic of Korea, during the whole study period. All monitors measured hourly data for all pollutants studied. We used 24-hr averages as the exposure index, by first averaging hourly values across all monitors for each day and then calculating 24-hr values. PM_10–2.5_ was calculated as PM_10_ minus PM_2.5_.

We calculated three types of long-term exposure: *a*) lifetime exposure, based on PM levels from birth to death or end of eligibility for outcome (1 year of age), *b*) gestational exposure (from conception to birth), and *c*) exposure for each trimester. We used length of gestation and birth date to estimate date of conception. Because data for exact day of birth were unavailable, we assigned birth day as the midpoint of the birth month. Trimesters were defined as 1–13 weeks, 14–26 weeks, and 27 weeks to birth; similar definitions have been applied elsewhere (Bell et al. 2007; Parker et al. 2005).

The National Meteorological Administration, Republic of Korea, provided hourly measurements of ambient temperature and 3-hr measurements of relative humidity for Seoul during the study period. We converted weather data into 24-hr values. To represent meteorologic conditions, we used the heat index, which is a function of temperature and relative humidity suggested by the U.S. National Weather Service (Rothfusz 1990):


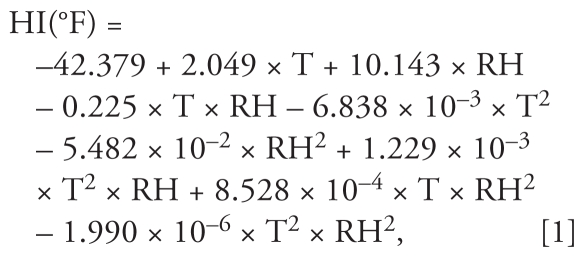


where HI is the heat index (°F), T is air temperature (°F), and RH is relative humidity (%). Variables for heat index, converted to degrees Celsius, were calculated for the gestation period, each trimester, and day of death in the lifetime exposure model. Similar control of weather variables was applied in previous models of air pollution exposure during pregnancy and infant outcomes (Bell et al. 2007). Data were available for 100% of days in the study period for all pollutants and weather variables.

### Statistical analysis

We used an extended Cox proportional hazards model with time-dependent covariates to investigate the association of PM with infant mortality. The Cox proportional hazards model has been widely used in analysis of time-to-event data with censoring and covariates (Fisher and Lin 1999). This standard Cox proportional hazards model assumes a constant hazard ratio over time. Thus, to estimate hazards with a varying exposure period, we used an extended Cox model with time-dependent covariates, which requires no proportional hazards, because the hazards depended on time, which in this case refers to study subjects’ age. An important assumption of the extended Cox model is that the effect of a time-dependent variable *X_i_*(*t*) on the survival probability at time *t* depends on the value of this variable at that same time *t*, and not on the value at an earlier or later time. The use of time-dependent survival methods ensures that effects are examined relative to other subjects for the same follow-up interval, similar to the manner in which matching is used in other analyses to establish comparability. This model has been applied previously (Platt et al. 2004; Suh et al. 2009).

**Figure 1:**
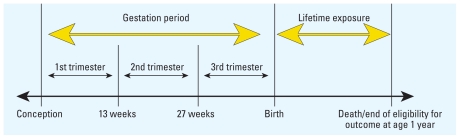
Structure of time-dependent variables for air pollutant exposure.

We analyzed separate models for each mortality type (e.g., respiratory causes) and pollutant (e.g., PM_2.5_). We stratified analysis by birth weight (normal birth weight, ≥ 2,500 g; low birth weight, < 2,500 g). We created time-dependent variables for cumulative lifetime exposure (from birth to death or end of eligibility for outcome at age 1 year) and for gestational exposure (for total pregnancy and each trimester) to each pollutant (TSP, PM_10_, PM_2.5_, and PM_10–2.5_) (Figure 1).

For each cause of death, air pollutant, birth weight group (normal or low birth weight), and exposure period (e.g., specific trimester), we fitted a time-dependent Cox proportional hazards model:


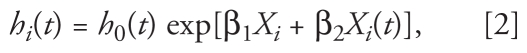


where *h*_0_(*t*) is the unspecified baseline hazard function, *X_i_* is the vector of time-independent variables (sex, length of gestation, season of birth, mother’s age, mother’s educational level, heat index), *X_i_*(*t*) is the vector of the time-dependent variable (PM), and β*_j_* (*j* = 1, 2) are vectors of model parameters.

Length of gestation was used as a continuous variable in weeks. Mother’s educational level was categorized as ≤ 6, 7–12, and > 12 years. To incorporate the nonlinear relationship between mother’s age and infant mortality, mother’s age was categorized as < 20, 20–24, 25–29, 30–34, 35–39, and > 39 years. Data on mother’s smoking status or alcohol use during pregnancy are not recorded on Korean birth certificates.

We performed a test of proportionality for time-fixed covariates with a few levels (e.g., mother’s educational level). For time-dependent covariates, we considered an extended Cox proportional hazards model with time-dependent covariates allowing nonproportional hazards because hazard ratios may vary across time. Analyses were conducted with SAS (version 9.2; SAS Institute Inc., Cary, NC, USA).

## Results

Table 1 shows characteristics of the study population, weather, and pollutant exposures. Analysis included 359,459 subjects, 2.0% with low birth weight. There were 225 total deaths, 26 SIDS deaths, and 22 respiratory-related deaths. Most respiratory deaths were from pneumonia and pulmonary disease (72.7%). The all-cause category included a small number of respiratory deaths (10%) and SIDS (12%). The remaining deaths are mainly composed of congenital malformations of the circulatory system (33.7%), other ill-defined and unspecified causes of mortality (20%), respiratory and cardiovascular disorders specific to the perinatal period (12.6%), and other forms of heart disease (10%). The characteristics of normal-birth-weight infants were similar to those of the subjects who died (all-cause mortality) for most factors except season of birth. Low-birth-weight infants were more likely to be female and to have a shorter gestation than were normal-birth-weight infants. Gestational PM concentrations were slightly higher among infants who died compared with all normal-birth-weight infants for all particle sizes (e.g., 31.5 vs. 30.6 μg/m^3^ for PM_2.5_). TSP, PM_10_, PM_2.5_, and PM_10–2.5_ gestational or lifetime exposures were similar across study subjects’ sex, gestational age, mother’s age, and mother’s education. Annual levels were similar across years for PM_10_, PM_2.5_, and PM_10–2.5_, whereas TSP decreased slightly over the study period (97.4 μg/m^3^ annual average in 2004, 92.6 μg/m^3^ in 2007).

As preliminary analysis, we investigated the relationship between infant mortality and covariates without including PM in the model (Table 2). Infant mortality was associated with lower birth weight, shorter gestation, older or younger mothers, and less maternal education.

First-, second-, and third-trimester exposures can be highly correlated (Bell et al. 2007), but in this study they were not (Pearson correlation coefficients: TSP, −0.10, −0.67, and −0.05, respectively; PM_10_, −0.11, −0.71, and −0.09; PM_2.5_, −0.07, −0.31, and −0.00; PM_10–2.5_, −0.12, −0.74, and −0.16). We performed analyses including all trimester exposures simultaneously and including each trimester separately. Results of both models were consistent.

Table 3 shows hazard ratios for all-cause infant mortality. We stratified by birth weight to assess potential susceptibility for low- and normal-birth-weight infants separately. We observed a relationship between gestational exposures and all-cause mortality for normal-birth-weight infants for all PM measures, but results for PM_10–2.5_ did not reach statistical significance. For low-birth-weight infants, we did not find a statistically significant relationship between gestational PM exposure and total mortality for any PM size; however, results should be viewed in the context of the smaller sample size (*n* = 7,054). For normal-birth-weight infants, the trimester of PM exposure with the highest effect estimate was the third for TSP, PM_10_, and PM_10–2.5_ and the first or second for PM_2.5_. The only significant association by trimester exposure was for first-trimester PM_2.5_. In the lifetime exposure model, we observed no statistically significant associations between any PM sizes and infant mortality from all causes for both birth weight groups, except for lower risk of mortality with higher PM_10–2.5_. We performed sensitivity analysis with lifetime exposure for heat index also included in the lifetime exposure model; results were similar (results not shown). Statistically significant results in Table 3 are robust to analysis of all-cause mortality excluding SIDS and respiratory-related deaths (results not shown).

We examined the association between long-term exposure to different particle sizes and cause-specific mortality for normal-birth-weight infants (Table 4). We found associations between gestational exposure to all PM sizes and respiratory infant mortality, although PM_10–2.5_ results did not reach statistical significance. Estimated risks per interquartile-range (IQR) increase in gestational exposure to TSP, PM_10_, and PM_2.5_ on infant respiratory-related mortality were 3.78 [95% confidence interval (CI), 1.18–12.13], 6.20 (95% CI, 1.50–25.66), and 3.15 (95% CI, 1.26–7.85), respectively. For comparability, we calculated trimester results in Table 4 for an increment of exposure based on the IQR for gestational exposure, representing the difference between 75th and 25th percentiles. The magnitude of risk for infant respiratory-related mortality was higher than for all causes (Tables 3 and 4). The most important trimester of exposure based on the highest central estimate was the first for TSP, PM_10_, PM_2.5_, and PM_10–2.5_, with statistically significant associations for all but PM_10–2.5_.

## Discussion

We conducted this study to estimate effects of long-term PM exposure on infant mortality in a birth cohort in Seoul, Korea, 2004–2007. We also evaluated which particle sizes are more related to all-cause and cause-specific infant mortality. We found statistically significant relationships between gestational PM exposures and infant mortality from all causes or respiratory causes for normal-birth-weight infants.

Results from epidemiologic studies should be interpreted in the context of the exposure increment used, which in our case was smaller than in some previous studies. For example, in earlier work on infant mortality, IQR PM_10_ values were 42.9 μg/m^3^ (Ha et al. 2003), 10 μg/m^3^ (Lipfert et al. 2000; Woodruff et al. 2008), 67 μg/m^3^ (Tsai et al. 2006), and 20 μg/m^3^ (Romieu et al. 2004), compared with our value of 6.93 μg/m^3^. IQR PM_2.5_ values were 10 μg/m^3^ (Loomis et al. 1999; Woodruff et al. 2006), whereas our value was 3.15 μg/m^3^.

The numbers of SIDS (*n* = 26) and respiratory (*n* = 22) deaths in this analysis are small. Despite the reduced statistical power, we found statistically significant associations between PM and respiratory-related infant mortality. The survival analysis approach provides more statistical power than case-crossover analysis because all study subjects are included; therefore, this approach provides benefits in the study of rare outcomes. However, additional analysis on the impact of air pollution during pregnancy and these rare mortality outcomes is warranted because the small number of deaths limited our ability to fully evaluate these findings. Such analysis could consider a larger spatial area and/or longer time frame, which would increase the number of mortality events.

Few studies have investigated long-term PM exposure and infant mortality. Below we discuss previous studies of short-term PM exposure and infant mortality that have the same health outcome as our study but different exposure periods (e.g., we include pregnancy exposures in our study, unlike previous studies). We then briefly summarize representative previous studies of air pollution and birth outcomes that have different health end points than our study but similar exposure periods (e.g., prenatal exposure). The biological mechanisms that link PM and pregnancy outcomes likely differ by health outcome or exposure time frame; however, these studies can provide context of the overall evidence of how PM affects infant health.

The relationship with gestational PM exposure was stronger for respiratory mortality than for all-cause mortality. Previous research also found higher effects for respiratory than for all-cause mortality for short-term postnatal PM exposure. A time-series study of postneonatal infant mortality in Korea provided an adjusted relative risk of 1.14 (95% CI, 1.10–1.19) for total mortality and 2.02 (1.78–2.28) for respiratory mortality per IQR (42.9 μg/m^3^) in same-day PM_10_ (Ha et al. 2003). Woodruff et al. (2006) found an adjusted odds ratio per 10-μg/m^3^ increase in PM_2.5_ lifetime exposure (infants’ birth to death) of 1.07 (95% CI, 0.93–1.24) for total mortality and 2.13 (1.12–4.05) for respiratory-related mortality. For all-cause mortality, our analysis found risks of 1.44 (95% CI, 1.06–1.97), 1.65 (1.18–2.31), and 1.53 (1.22–1.90) per IQR increase (8.91, 6.93, 3.15 μg/m^3^) in gestational exposure to TSP, PM_10_, and PM_2.5_, respectively, and for respiratory mortality we found risks of 3.78 (1.18–12.13), 6.20 (1.50–25.66), and 3.15 (1.26–7.85).

We did not find statistically significant relationships between any PM size and SIDS. Previous studies found inconsistent results for this outcome. Woodruff et al. (1997) found a statistically significant relationship between PM_10_ during the first 2 months of life and SIDS. Another study in California did not find a relationship between PM_2.5_ from birth to death and SIDS (Woodruff et al. 2006). A recent U.S. study examining PM_2.5_, PM_10_, ozone, sulfur dioxide, and carbon monoxide during the first 2 months of life found that ozone may be associated with SIDS but did not observe PM effects (Woodruff et al. 2008). Further analysis is needed to investigate possible links between PM and SIDS.

We observed a positive effect on infant mortality in some lifetime exposure models (e.g., PM_10–2.5_ and normal-birth-weight infants for all-cause mortality). We controlled for weather and season of birth, although other temporally varying confounders may exist. Our adjustment for several maternal and individual factors that are unlikely to vary temporally are unlikely to influence results because this study focuses on a single city; however, similar studies in larger geographic areas should carefully consider such potential confounders. Our use of individual information on mother’s education as an indicator of socioeconomic status may not fully reflect full socioeconomic conditions. Actual socioeconomic status is also related to the educational level of other members of the household and many other factors such as income, occupation, housing type, the type and age of vehicles, and the air conditioning system at the residence. Further, socioeconomic conditions are a function of previous history of socioeconomic conditions such as previous income. Recent literature showed that the neighborhood environment of the mother and child has an independent influence on birth outcomes that was not explained by individual-level risk factors (Buka et al. 2003; O’Neill et al. 2003). In previous studies in Korea, area-level socioeconomic status modified the association between air pollution and health outcomes (Lee et al. 2006; Yi et al. 2010). Further study is needed to examine potential confounding and effect modification by socioeconomic status and to investigate the associations observed in this study.

Many studies suggest that air pollution exposure during pregnancy is associated with birth outcomes such as preterm birth, low birth weight, intrauterine growth restriction, and birth defects (Ballester et al. 2010; Pope et al. 2010; Ritz et al. 2002; Tsai et al. 2003). Although these studies have exposure time frames similar to those in our study, the health outcomes differ, as may the biological mechanisms. One study found a 16% increase in risk of preterm birth per 50 μg/m^3^ in PM_10_ during the first month of pregnancy (Ritz et al. 2000). The relative risk for small for gestational age was 1.03 per 1-μg/m^3^ increase in PM_2.5_ in the second trimester, with a corresponding birth weight reduction of 4.1 g (95% CI, 1.4–6.8 g) (Mannes et al. 2005). Another study found that an IQR increase in gestational exposure to PM_10_ and PM_2.5_ lowered birth weight by 8.2 g (5.3–11.1 g) and 14.7 g (12.3–17.1 g), respectively (Bell et al. 2007).

Low birth weight may have an important effect on infant health and is a significant determinant of infant mortality (McCormick 1985). Low birth weight is mainly caused by preterm birth, defined as delivery < 37 weeks of pregnancy, and fetal growth retardation (Xu et al. 2011). Many studies showed that crucial factors of infant mortality, such as low birth weight and gestational age, are associated with increased infant mortality (Callaghan et al. 2006; Donovan et al. 2010; Were and Bwibo 2009). In a study in Korea, exposure to air pollution during pregnancy was significantly associated with preterm birth (Ha et al. 2004) and low birth weight (Ha et al. 2001; Lee et al. 2003).

Prenatal exposures may enhance susceptibility and increase risk of infant mortality. Although multiple mechanisms have been proposed, the biological pathways by which PM could affect infant mortality are not fully understood. Air pollution could directly affect fetal growth through the placenta or indirectly by impairing mother’s health (Glinianaia et al. 2004b). PM is known to invoke an inflammatory response that alters blood coagulation, invoke an allergic immune response, and alter cardiac function by reducing heart rate variability (Gold et al. 2000; Liao et al. 1999; Seaton et al. 1995). All these mechanisms can occur in the fetus as well as in adults. Also, maternal exposure to PM air pollutants during pregnancy can result in reduced transplacental function, with consequent deterioration in fetal growth and development (Glinianaia et al. 2004b). The biological mechanism through which exposure to air pollution affects risk of mortality may vary by several factors, including the pollutant (e.g., chemical structure of the PM mixture), time frame of exposure (e.g., exposure during pregnancy vs. after birth), and cause of mortality. As research continues to develop our understanding of the physiologic pathways through which air pollution affects risk, future work may examine alternate model structures that incorporate this information, such as the most relevant period of exposure for the heat index or related weather variables.

Because of the structure of the available data, we assumed birth day as the day representing the midpoint of the birth month and estimated time frame for exposure based on this information. Similar approaches have been used elsewhere (Bell et al. 2007). Although this is a limitation of our study, it does not greatly affect long-term exposure estimates. The estimated date of birth cannot be > 2 weeks from the actual date of birth. Such an approach would be problematic for study of acute exposures, although for gestational exposure this approach can result in a maximum of 5% of exposure based on the incorrect time frame.

In this study, we controlled for covariates such as individual and maternal characteristics (Table 2). Our analysis showed that the infants having shorter gestation periods might be at a greater risk for infant mortality. We found that the risk of infant mortality was associated with lower educational level of mother, lower birth weight, and older or younger maternal age, which is consistent with previous research. In a study by Arntzen et al. (2004), the risk of infant mortality decreased with higher educational attainment. Gnavi and Costa (2002) reported that postneonatal mortality was strongly correlated with low maternal educational level. Adair and Popkin (1988) observed that low-birth-weight infants had a higher risk of infant mortality. We were not able to control for maternal smoking, because these data are not available on the Korean birth certificates. However, this factor is unlikely to significantly alter the effect estimates, because smoking among woman in Korea is not highly prevalent, particularly among pregnant women. The smoking level of women in Korea is the lowest (4.6%) among many countries (Organisation for Economic Cooperation and Development 2009).

Our study differs from previous work in several ways. We focused on long-term exposure and applied a survival analysis with time-dependent covariates. Many epidemiologic studies have examined associations between air pollution and infant mortality by using time-series or case-crossover studies to estimate the effects of short-term exposure to air pollution. In the time-series studies, individual data on the subjects often are not available, which limits the assessment of individual risk factors. Our analytical framework enables identification and adjustment for individual risk factors and increases the statistical power because all the subjects (deceased and alive) are included. Lepeule et al. (2006), who reported an association between air pollution and cardiorespiratory mortality, found that the results using survival analysis agreed with those obtained with the case-crossover analysis and that confidence intervals are more restricted with the Cox proportional hazards model. A study by Platt et al. (2004) supported the effectiveness of applying the extended Cox proportional hazards model for the study of fetal and infant death.

To our knowledge, this is the first study to investigate the association between long-term exposure to different sizes of particles and infant mortality in Korea. Our findings provide evidence supporting the hypothesis that long-term exposure to PM air pollution during pregnancy increases the risk of infant mortality from all causes or respiratory causes. We also suggest that using the Cox proportional hazard model with time-dependent covariates to examine the effect of air pollution in a cohort study with a time-dependent exposure has benefits over more traditionally applied approaches.

## Figures and Tables

**Table 1:** Characteristics for the birth cohort in Seoul, Korea, 2004–2007.

Characteristic	Eligible births (*n* = 359,459)		Total mortality (all causes; *n* = 225)
	Normal birth weight (*n* = 352,405)	Low birth weight (*n* = 7,054)	
Sex (%)			
Male	51.6	40.8	52.0
Female	48.4	59.2	48.0
Age at death [years (mean ± SD)]	—	—	0.40 ± 0.25
Birth weight [kg (mean ± SD)]	3.31 ± 0.38	2.30 ± 0.18	3.13 ± 0.49
Gestational age [weeks (mean ± SD)]	39.3 ± 1.1	38.1 ± 1.1	39.0 ± 1.1
37–38 weeks (%)	25.2	67.5	38.7
39–40 weeks (%)	63.4	30.5	54.7
41–42 weeks (%)	11.3	2.0	6.6
43–44 weeks (%)	0.1	0.0	0.00
Mother’s age (%)			
< 20 years	0.2	0.4	1.3
20–24 years	4.3	4.4	5.3
25–29 years	36.3	32.5	34.2
30–34 years	46.7	47.0	45.8
35–39 years	11.2	13.8	12.0
> 39 years	1.3	1.9	1.3
Educational level of mother [years (%)]			
≤ 6	0.3	0.5	0.4
7–12	33.2	35.4	39.6
> 12	66.5	64.1	60.0
Season of birth (%)			
Winter	25.1	24.2	26.2
Spring	25.7	25.9	26.7
Summer	23.7	24.1	27.6
Fall	25.5	25.8	19.5
Pollution exposures during pregnancy [μg/m^3^ (mean ± SD)]			
TSP			
Gestation	96.7 ± 6.6	96.3 ± 7.2	98.0 ± 6.5
First trimester	94.8 ± 18.6	94.1 ± 18.6	94.9 ± 18.3
Second trimester	93.1 ± 18.2	93.3 ± 18.3	95.3 ± 17.5
Third trimester	93.1 ± 20.1	92.8 ± 21.0	98.3 ± 19.2
PM_10_			
Gestation	61.3 ± 4.2	61.2 ± 4.6	62.2 ± 4.4
First trimester	61.7 ± 12.0	61.4 ± 12.0	61.1 ± 11.8
Second trimester	60.5 ± 11.7	60.6 ± 11.8	61.0 ± 11.0
Third trimester	60.7 ± 12.7	60.6 ± 13.4	62.9 ± 12.0
PM_2.5_			
Gestation	30.6 ± 2.3	30.5 ± 2.3	31.5 ± 2.5
First trimester	31.1 ± 5.1	30.9 ± 4.9	31.7 ± 5.8
Second trimester	30.1 ± 4.1	30.1 ± 4.1	30.5 ± 4.0
Third trimester	30.2 ± 4.6	30.1 ± 4.9	31.1 ± 4.5
PM_10–2.5_			
Gestation	30.6 ± 2.6	30.6 ± 2.9	30.7 ± 2.7
First trimester	30.6 ± 8.1	30.4 ± 8.1	29.4 ± 7.5
Second trimester	30.4 ± 8.2	30.5 ± 8.2	30.5 ± 7.7
Third trimester	30.5 ± 8.6	30.5 ± 9.0	31.8 ± 8.2
Lifetime pollution exposure [μg/m^3^ (mean ± SD)]			
TSP	88.7 ± 14.4	88.8 ± 14.7	91.7 ± 18.5
PM_10_	59.5 ± 5.7	59.5 ± 6.0	59.1 ± 11.6
PM_2.5_	29.1 ± 1.7	29.1 ± 1.9	29.5 ± 4.6
PM_10–2.5_	30.5 ± 4.1	30.5 ± 4.2	29.7 ± 7.5

**Table 2:** Hazard ratios for all-cause infant mortality associated with selected nonpollution variables.

Variable	Hazard ratio (95% CI)
Birth weight (kg)	0.54 (0.36–0.80)
Child’s sex	
Male	Reference
Female	0.91 (0.69–1.19)
Gestational length (weeks)	0.85 (0.75–0.96)
Mother’s age (years)	
< 20	Reference
20–24	0.19 (0.05–0.69)
25–29	0.19 (0.06–0.60)
30–34	0.19 (0.06–0.62)
35–39	0.21 (0.06–0.72)
> 39	0.21 (0.04–1.02)
Mother’s education (years)	
≤ 6	Reference
7–12	0.75 (0.10–5.46)
> 12	0.63 (0.09–4.64)
Season of birth	
Winter	1.07 (0.74–1.55)
Spring	Reference
Summer	1.13 (0.78–1.64)
Fall	0.83 (0.55–1.25)

**Table 3:** Hazard ratios for an IQR increase from Cox proportional hazards models^a^ on all-cause infant mortality in a birth cohort, Seoul, Korea, 2004–2007.

Exposure	Hazard ratio (95% CI)	
	Normal birth weight (*n* = 352,405)	Low birth weight (*n* = 7,054)
Gestational exposure		
TSP (IQR, 8.91 μg/m^3^)		
All	1.44 (1.06–1.97)	1.69 (0.38–7.49)
First trimester	1.03 (0.92–1.16)	1.13 (0.69–1.85)
Second trimester	1.03 (0.91–1.16)	1.40 (0.89–2.18)
Third trimester	1.10 (0.98–1.23)	0.80 (0.54–1.19)
PM_10_ (IQR, 6.93 μg/m^3^)		
All	1.65 (1.18–2.31)	1.48 (0.38–5.80)
First trimester	1.06 (0.93–1.22)	1.13 (0.67–1.89)
Second trimester	1.04 (0.89–1.20)	1.43 (0.85–2.41)
Third trimester	1.07 (0.93–1.23)	0.80 (0.50–1.28)
PM_2.5_ (IQR, 3.15 μg/m^3^)		
All	1.53 (1.22–1.90)	1.00 (0.34–2.94)
First trimester	1.15 (1.04–1.28)	1.03 (0.63–1.69)
Second trimester	1.15 (0.96–1.38)	1.27 (0.62–2.58)
Third trimester	1.06 (0.89–1.27)	0.85 (0.47–1.54)
PM_10–2.5_ (IQR, 3.71 μg/m^3^)		
All	1.19 (0.83–1.70)	1.92 (0.49–7.63)
First trimester	0.94 (0.84–1.05)	1.13 (0.76–1.69)
Second trimester	0.99 (0.89–1.11)	1.30 (0.90–1.86)
Third trimester	1.05 (0.95–1.16)	0.84 (0.58–1.21)

Lifetime exposure		
TSP (IQR, 18.99 μg/m^3^)	1.01 (0.75–1.36)	0.91 (0.25–3.28)
PM_10_ (IQR, 3.48 μg/m^3^)	0.94 (0.87–1.02)	0.93 (0.67–1.30)
PM_2.5_ (IQR, 1.25 μg/m^3^)	1.00 (0.93–1.08)	1.08 (0.83–1.42)
PM_10–2.5_ (IQR, 2.01 μg/m^3^)	0.92 (0.85–0.99)	0.81 (0.59–1.12)

The 352,405 infants of normal birth weight in this analysis include 209 all-cause deaths. The 7,054 infants of low birth weight include 16 all-cause deaths.

a The model included the following variables: sex, gestation period, educational level of mother, maternal age, season of birth, and heat index.

**Table 4:** Hazard ratios for an IQR increase from Cox proportional hazards models^a^ on cause-specific infant mortality for normal-birth-weight infants in a birth cohort, Seoul, Korea, 2004–2007.

Exposure	Hazard ratio (95% CI)	
	Respiratory	SIDS
Gestational exposure		
TSP (IQR, 8.91 μg/m^3^)		
All	3.78 (1.18–12.13)	0.92 (0.33–2.58)
First trimester	2.08 (1.26–3.43)	1.02 (0.72–1.45)
Second trimester	0.96 (0.64–1.45)	0.80 (0.58–1.10)
Third trimester	1.03 (0.71–1.48)	1.00 (0.74–1.37)
PM_10_ (IQR, 6.93 μg/m^3^)		
All	6.20 (1.50–25.66)	1.15 (0.38–3.48)
First trimester	2.19 (1.30–3.70)	1.04 (0.70–1.55)
Second trimester	0.97 (0.59–1.60)	0.79 (0.53–1.17)
Third trimester	1.04 (0.66–1.64)	0.95 (0.64–1.41)
PM_2.5_ (IQR, 3.15 μg/m^3^)		
All	3.15 (1.26–7.85)	1.42 (0.71–2.87)
First trimester	1.58 (1.14–2.19)	1.14 (0.82–1.59)
Second trimester	1.09 (0.61–1.93)	0.89 (0.54–1.47)
Third trimester	1.46 (0.79–2.68)	0.80 (0.48–1.35)
PM_10–2.5_ (IQR, 3.71 μg/m^3^)		
All	2.86 (0.76–10.85)	0.57 (0.16–1.96)
First trimester	1.45 (0.99–2.14)	0.95 (0.69–1.31)
Second trimester	0.94 (0.63–1.39)	0.81 (0.60–1.10)
Third trimester	0.93 (0.66–1.32)	1.01 (0.76–1.34)

Lifetime exposure		
TSP (IQR, 18.99 μg/m^3^)	0.35 (0.07–1.86)	0.81 (0.33–2.00)
PM_10_ (IQR, 3.48 μg/m^3^)	0.65 (0.43–0.99)	0.73 (0.57–0.94)
PM_2.5_ (IQR, 1.25 μg/m^3^)	0.63 (0.42–0.95)	0.88 (0.71–1.09)
PM_10–2.5_ (IQR, 2.01 μg/m^3^)	0.70 (0.48–1.01)	0.66 (0.51–0.86)

The 352,405 infants of normal birth weight in this analysis include 22 respiratory deaths and 26 SIDS deaths.

a The model included the following variables: sex, gestation period, educational level of mother, maternal age, season of birth, and heat index.
